# Live Imaging of Whole Mouse Embryos during Gastrulation: Migration Analyses of Epiblast and Mesodermal Cells

**DOI:** 10.1371/journal.pone.0064506

**Published:** 2013-07-08

**Authors:** Takehiko Ichikawa, Kenichi Nakazato, Philipp J. Keller, Hiroko Kajiura-Kobayashi, Ernst H. K. Stelzer, Atsushi Mochizuki, Shigenori Nonaka

**Affiliations:** 1 Laboratory for Spatiotemporal Regulations, National Institute for Basic Biology, Okazaki Aichi, Japan; 2 Theoretical Biology Laboratory, RIKEN Advanced Science Institute, Wako-city, Saitama, Japan; 3 Janelia Farm Research Campus, Howard Hughes Medical Institute, Ashburn, Virginia, United States of America; 4 Physical Biology (FB 15 IZN), Buchmann Institute for Molecular Life Sciences (BMLS, CEF-MC), Goethe Universität Frankfurt, Frankfurt am Main, Germany; 5 Department of Basic Biology, School of Life Science, The Graduate University for Advanced Studies (SOKENDAI), Hayama, Kanagawa, Japan; Institute of Science and Technology Austria, Austria

## Abstract

During gastrulation in the mouse embryo, dynamic cell movements including epiblast invagination and mesodermal layer expansion lead to the establishment of the three-layered body plan. The precise details of these movements, however, are sometimes elusive, because of the limitations in live imaging. To overcome this problem, we developed techniques to enable observation of living mouse embryos with digital scanned light sheet microscope (DSLM). The achieved deep and high time-resolution images of GFP-expressing nuclei and following 3D tracking analysis revealed the following findings: (*i*) Interkinetic nuclear migration (INM) occurs in the epiblast at embryonic day (E)6 and 6.5. (*ii*) INM-like migration occurs in the E5.5 embryo, when the epiblast is a monolayer and not yet pseudostratified. (*iii*) Primary driving force for INM at E6.5 is not pressure from neighboring nuclei. (*iv*) Mesodermal cells migrate not as a sheet but as individual cells without coordination.

## Introduction

Establishment of the three germ layers during gastrulation occurs via a highly orchestrated set of morphogenetic events that shape the early embryo [1]–[5]. In mouse embryos, this process begins as epiblast cells traverse the primitive streak at the posterior end. These cells then differentiate into mesodermal cells and migrate anteriorly. Although these events are essential for subsequent embryonic morphogenesis, live imaging of these processes has been strictly limited to a part of the embryo [6], [7].

Live imaging analysis of whole mouse embryos during gastrulation requires deep optical penetration of the specimen and high time resolution. Conventional wide-field fluorescence microscopes suffer from low contrast, and confocal fluorescence microscopy permits only superficial visualization of the embryo. Multi-photon microscopy allows imaging to a greater depth, but the temporal resolution is not sufficient for cell tracking. In order to overcome these limitations, we used digital scanned light-sheet microscopy (DSLM) [8], [9], a type of light sheet-based fluorescence microscopy (LSFM) that uses planar illumination perpendicular to the detection axis (Figure S1). This method offers the advantages of a high signal-to-noise ratio (S/N), high speed, and good optical penetration [10], [11]. In this study, we used DSLM in combination with a mouse embryo culture system to perform time-lapse imaging of whole mouse embryos during gastrulation. We also developed software tools for tracking of the nuclei, and report cell migration properties of the epiblast and the mesodermal cells.

## Results

### Mouse embryo culture system for DSLM

**Figure 1:**
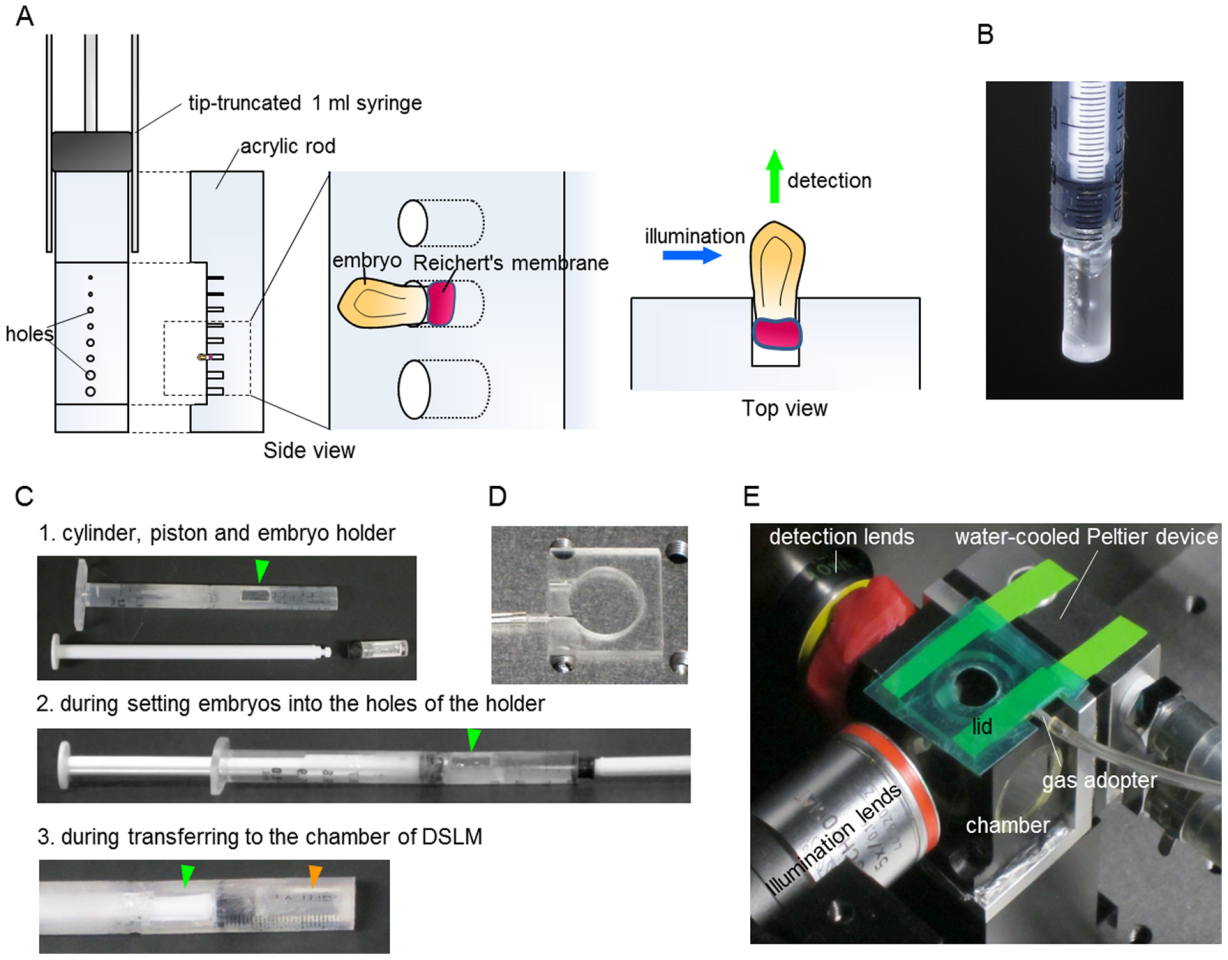
Mouse embryo culture system for DSLM. (A) Side and top views of the embryo holder. The embryos are placed into the holes of an acrylic rod attached to the piston of a tip-truncated 1-ml syringe. The embryo is held stably in the hole via the Reichert's membrane at the ectoplacental cone. (B) Photograph of the embryo holder. (C) The embryo transfer method. For embryo transfer from dish to the chamber of the DSLM, we use the cylinder with window (window is indicated by green arrowhead). Embryos are moved to the space of the holder filled with culture medium under wide-field microscope (middle). After setting embryo into the holder, the holder is pushed until the end of the cylinder and stayed at the position without window (bottom, the position of the holder is indicated by orange arrowhead). At this position we can move the holder without loss of the culture medium. The holder is pushed into culture medium in the chamber of DSLM. (D) Photograph of the gas adopter. (E) Photograph of the assembled system around the chamber. The gas mixture is injected at the top of the chamber though the gas adopter. To prevent evaporation of the culture medium, a lid is set at the top of the adopter.

In order to observe a whole mouse embryo during gastrulation, we developed a series of techniques for mouse embryo culture in the chamber of DSLM. These techniques include a specific embryo holder for mouse embryos, transfer methods of embryos without exposing to the air, temperature regulation and an atmosphere control (Figure 1). Since mouse embryos cannot be cultured in the sort of conventional agarose holder typically used for samples in LSFM, we made a specimen holder for mouse embryos (Figure 1A and B). The embryos are placed onto the holes of an acrylic rod attached to the piston of a tip-truncated 1-ml syringe. The embryo is held stably in the hole via sticky Reichert's membrane at the ectoplacental cone. The embryo is transferred to the holder via a window on the cylinder (Figure 1C), and then set to the stage of the DSLM. This procedure prevents the embryos getting damaged by exposure to the air. For temperature control, we developed water-cooled Peltier device-mounted chamber (Figure 1D, HAYASHI WATCH-WORKS CO., LTD). The 5% CO_2_ and 5% O_2_ gas mixture is injected via a gas adopter into the specimen chamber, over the surface of culture medium (Figure 1E). A lid is put at the top of the chamber to prevent drying of culture medium.

### Live imaging of the whole mouse embryo at E6.5 by DSLM

**Figure 2:**
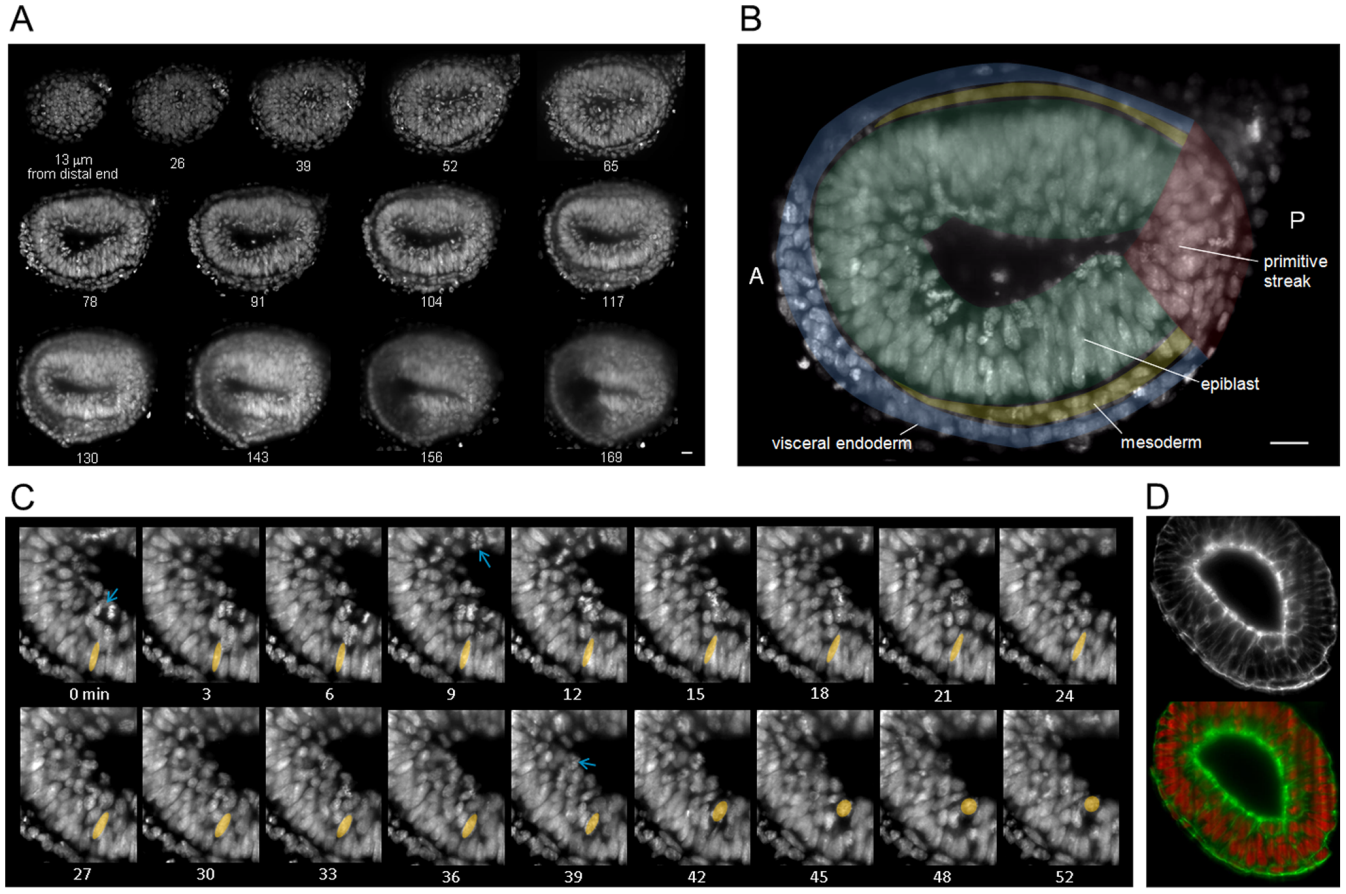
Live imaging of the whole mouse embryo at E6.5 using DSLM. (A) Optical sections of a Histone H2B-GFP mouse embryo along the axis perpendicular to the proximal–distal axis. Each image represents the maximum-intensity projection of a 13-µm thick stack. (B) Annotated slice is 78 µm from the distal end of the embryo. Blue, visceral endoderm; green, epiblast; yellow, mesoderm; red, primitive streak. Anterior (A) is to the left, posterior (P) to the right. Scale bar  = 20 µm. (C) Time series of a magnified region of the epiblast. Blue arrows indicate dividing nuclei; the colored nucleus exhibits interkinetic nuclear migration in the apical direction interkinetic nuclear migration in the apical direction (Movie S1). (D) Image of a section stained with Alexa Fluor 546 phalloidin (membrane) and DRAQ5 (nucleus). Top panel: membrane marker; bottom panel: overlay of membrane and nucleus markers.

We then performed time-lapse imaging of Histone H2B-GFP–expressing mouse embryos at embryonic day 6.5 (E6.5) (Figure 2A–C and Movie S1). We obtained images from the distal end to the end of embryonic body with 1.5 minute interval, which is difficult with the other kind of microscopy. At this nearly maximum time resolution, the embryos exhibited abnormal development after ten hours of illumination (total energy, 46 mJ) although DSLM illuminates lower energy to the sample than the conventional microscopy, suggesting high fragility to phototoxicity of mouse embryos at this stage. Until three hours they looked normal without major developmental defects (Figure S2).

We observed nuclei in the epiblast migrate along the apical-basal axis and divide near the apical surface (Figure 2C). This phenomenon is known as interkinetic nuclear migration (INM) that occur in the neuroepithelium at later stages in mouse, chick, zebrafish, etc. [12]–[14] with cell cycle-dependent manner [15]. In E6.5 mouse epiblast, nuclei in S phase are reported to localize at basal side in fixed embryo [16]. INM has been observed in pseudostratified epithelium of the spinal cord, cerebral cortex, and retina [17]–[20], and in other pseudostratified tissues [21]–[23]. We confirmed the epiblast in this stage is pseudostratified by membrane staining of a fixed sample (Figure 2D), i.e. the INM in E6.5 epiblast appeared to be a common feature in pseudostratified epithelia.

### Live imaging of an E5.5 and E6 mouse embryos

**Figure 3:**
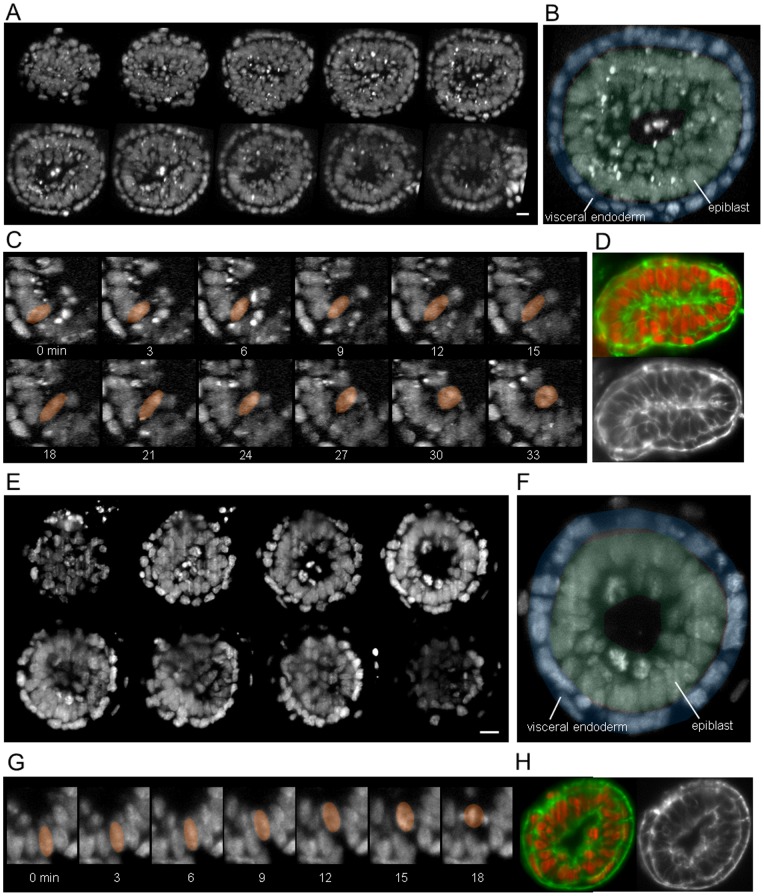
Live imaging of the whole mouse embryo at E6 (A to D) and E5.5. (E to H). (A and E) Each image represents the maximum intensity projection of a 13-µm thick section. Scale bar  = 20 µm. (B and F) Annotated sections are 65 (B) and 40 (E) µm from the distal end of the embryo. Blue and green areas indicate visceral endoderm and epiblast, respectively. (C and G) INM and INM-like movement in the epiblast. The colored nucleus exhibits apical migration. (D and H) Image of sections stained with Alexa Fluor 546 phalloidin (membrane) and DRAQ5 (nucleus).

Next we examined earlier stages, E5.5 and E6 (Figure 3). At E6, the structure of the epiblast is pseudostratified (Figure 3D), and exhibits INM as at E6.5 (Figure 3C and Movie S2). On the other hand, at E5.5 the epiblast does not yet appear to have a pseudostratified structure (Figure 3H) whereas the nuclei exhibit an INM-like movement (Figure 3G and Movie S3). This result was unexpected because INM is reported to be a conserved feature in pseudostratified structures [24]. Although the role of this INM-like movement is still unknown, we infer that there might be the relationship between INM and the transformation from a monolayer to a pseudostratified structure.

### Tracking the epiblast nuclei and characterization of INM

**Figure 4:**
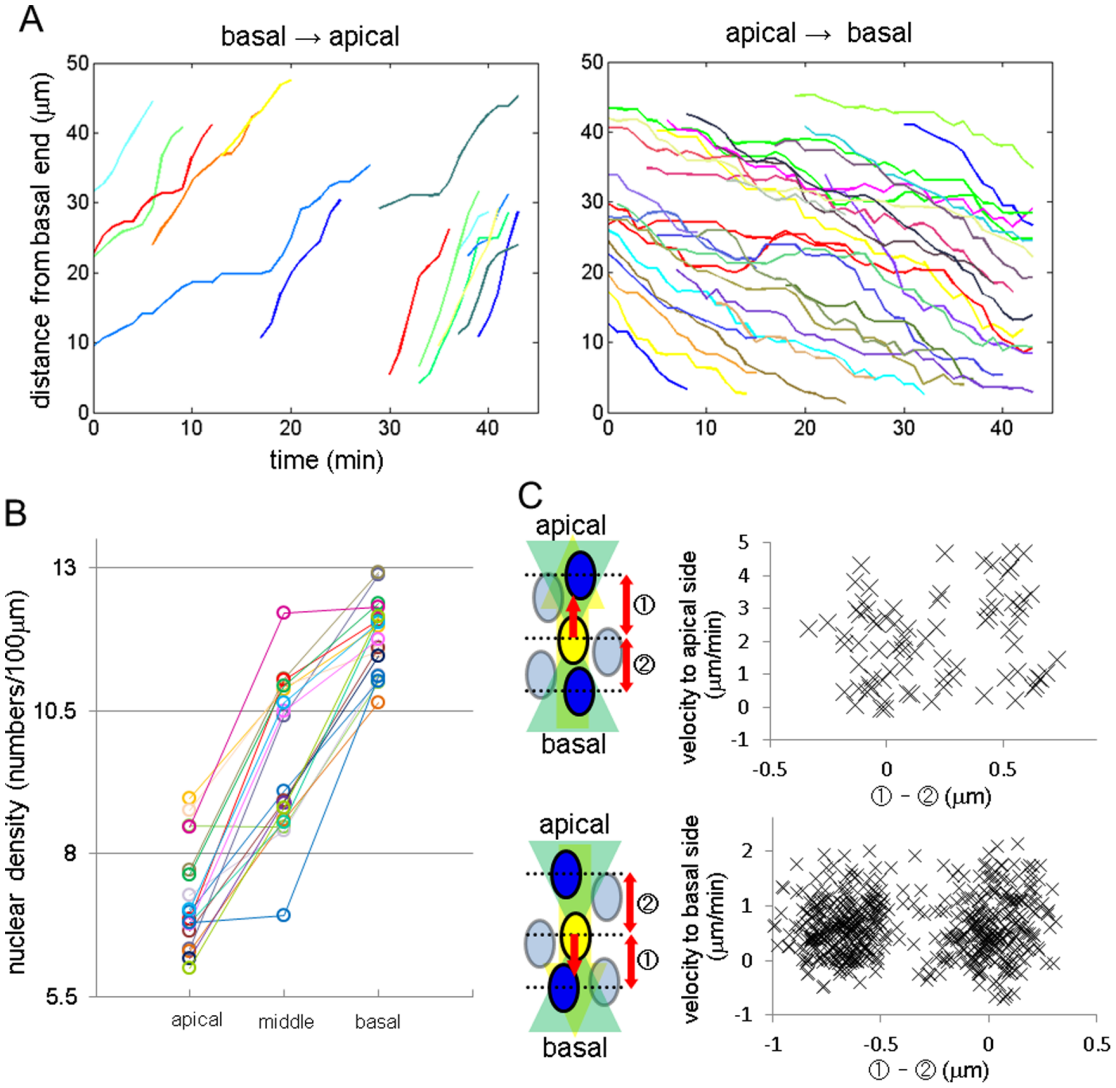
Three-dimensional tracking of all epiblast nuclei in a region of the epiblast. (A) Kymographs of apical and basal migrations. The positions of each nucleus were reconstructed in Movies S4 (distal view) and S5 (lateral view). (B) Nuclear density compared among three sections along apical–basal axis (n = 18). (C) The correlation between velocity and the difference between the distances of a nucleus from its forward and backward neighbors. If migrations were caused by pushing forces from neighboring nuclei, the velocity should become grater when the difference in distances increases. However, velocity in neither direction was correlated with the difference in distance (apical, R^2^ = 0.0232; basal, R^2^ = 0.0011).

To characterize INM, we tracked the three-dimensional (3D) positions of all nuclei in a portion of the epiblast at E6.5 (n = 103; 83 (anterior–posterior) ×42 (left–right) ×77 (proximal–distal) µm, Movie S4 and 5). Tracking was performed manually by the aid of a custom-made 3D tracking assistance software. Kymographs of both apical and basal nuclear migrations are shown in Figure 4A. We measured average speeds of 1.09±0.34 (mean ± SD) µm/min from the basal to the apical surface (n = 29) and 0.54±0.14 µm/min from the apical to the basal surface (n = 28). These results are consistent with the values measured in other tissues and species 25–27].

A recent study suggested that basal migration in mouse brain is not the result of a cell-autonomous force derived from intrinsic motor proteins, but rather of a passive pushing force caused by the apical migration of neighboring nuclei [28]. To evaluate the contribution of such force for the INM of mouse epiblast, we first measured the nuclear density at the apical, middle, and basal levels (Figure 4B). The density at the basal level is higher than that at the apical level (1.6×, n = 18), so it seems unlikely that the basal migration is caused passively by a pushing force from apically positioned nuclei. On the other hand, it is possible that basally positioned nuclei may create a force that pushes another nucleus apically. We next measured the correlation between the velocity of a nucleus and its relative position to neighboring nuclei. The latter was represented as the difference between the distance of a nucleus from its forwardly-positioned neighbor (i.e., the nucleus ahead of the cell in the direction of migration) and the distance from its backwardly-positioned neighbor (Figure 4C). If the migration is caused by a pushing force from backward-neighbor nuclei, the velocity should become greater as the difference in distances increases, indicating contact between the backward neighbor and the free forward space. The velocity of neither orientation was not correlated with the difference in distance (apical, R^2^ = 0.0232; basal, R^2^ = 0.0011). Therefore, in the epiblast of mouse embryo, the main driving force for both apical and basal nuclear migrations would be caused by cell-autonomous mechanisms, not by a passive force, although additional analysis is necessary for conclusion.

In order to further characterize INM in the epiblast at E6.5, we next evaluated the 3D orientation of cell division (Figure S3). The distribution of orientation of cell division had no apparent bias within the epiblast (Figure S3A). And nuclear divisions perpendicular to the apical surface occurred more often than those with oblique or parallel orientations (41% of total divisions, Figure S3B). This result is consistent with results obtained in fixed samples [16].

### Three-dimensional tracking of mesodermal cell migrations

**Figure 5:**
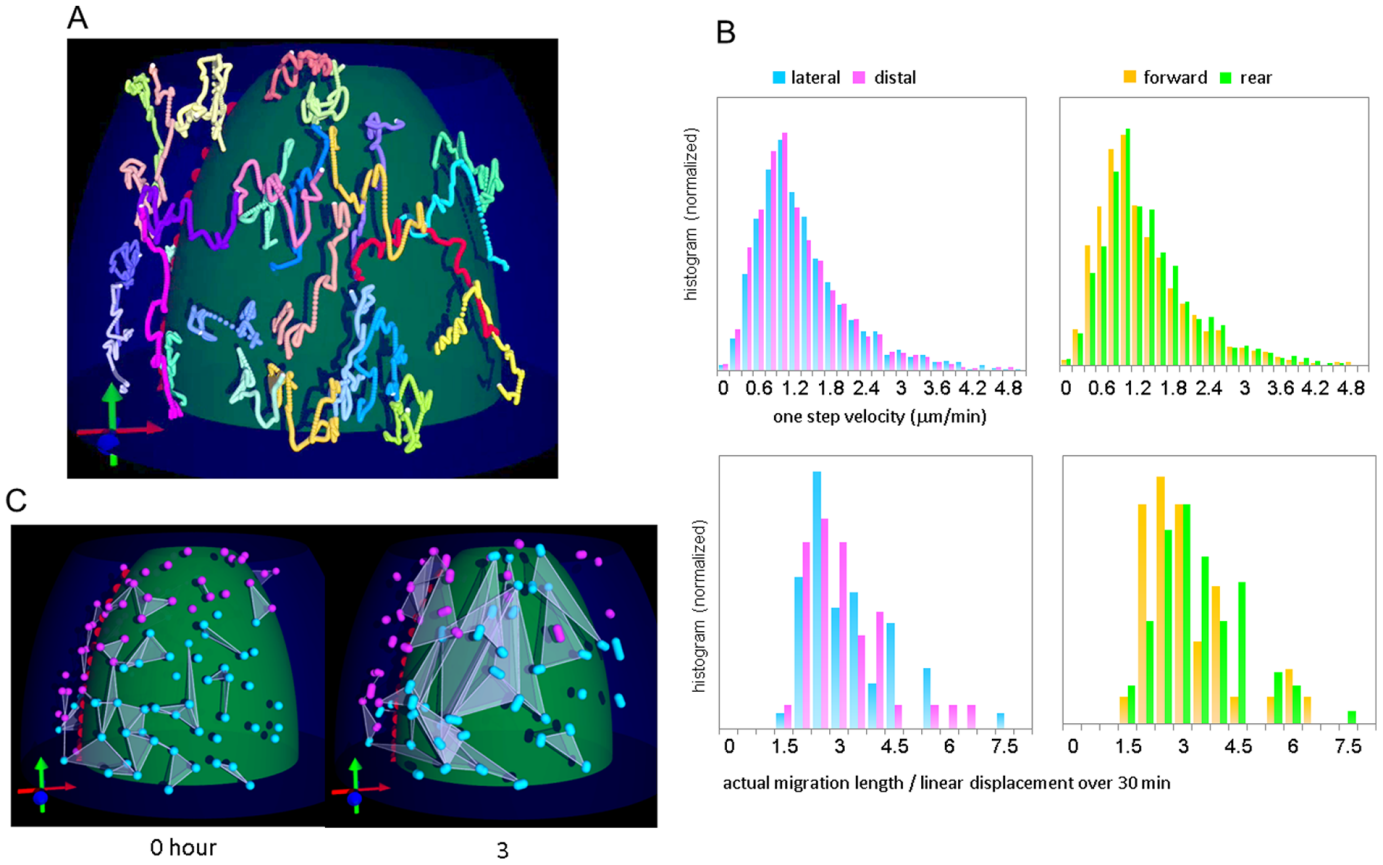
Three-dimensional tracking of mesodermal nuclei. (A) Computationally reconstructed trajectories (Movie S6 and S7). White mark at the end of trajectory indicates the latest point. Red, blue, and green arrows at the lower left indicate the anterior–posterior axis, right–left axis, and proximal–distal axis, respectively. (B) Histograms of one-step velocity (top row) and the ratio of actual migration path length to linear displacement over 30 min (bottom row). Nuclear positions at the first time-point were classified into two areas according to two sets of criteria, lateral (cyan) or distal (magenta) and forward (orange) or rear (green). (C) Comparison of triangles connected neighboring nuclei at first time point and after 3 hours (Movie S8). Nuclear positions at the first time-point were classified into two areas, lateral (cyan) or distal (magenta). After 3 hours, the areas of the triangles become larger and some triangles overlap each other, indicating that nuclei in the mesoderm do not migrate in straight lines.

Imaging by DSLM also allowed visualization of migrating mesodermal cells. In mouse embryo, time-lapse imaging of mesodermal cell has not been previously performed with high temporal and spatial resolution, hence the precise details of mesodermal cell migration have remained unknown [3]. In contrast to epiblast cells with elongated shape, mesodermal cells are compact so that movement the nuclei can be regarded as that of cells themselves. Migration of mesodermal nuclei was tracked using automatic tracking tools we developed. Reconstructed 3D trajectories are shown in Figure 5A and Movie S6. This result indicates that the paths followed by mesodermal cells are not straightforward. In order to elucidate these paths, we measured the distributions of the one-step velocities (top row in Figure 5B) and the ratio of the actual migration path length to the linear displacement (bottom row); this ratio indicates the degree of zig-zag movement. Previous reports predict that axial mesoderm cells located in the distal region at midline migrate in concert with the adjacent paraxial mesoderm [29]–[33], and that the cells in the forward mesoderm migrate more rapidly than cells elsewhere in the mesoderm [34]. We therefore separately measured nuclear positions in the lateral (cyan in Figure 5B) and axial (magenta) areas at the first time-point. We also classified nuclear positions as ‘forward’ (orange) and ‘rear’ (green). Figure 5C and Movie S8 demonstrate that triangle shapes connected neighboring nuclei at the first time-point intersect each other after three hours. These results show that the mesodermal cells migrate not as a coordinated group, but rather individually, *i.e*., that mesodermal cells in the mouse embryo do not maintain cell–cell adhesion as they do in *Xenopus* or Zebrafish mesendoderm [35]–[38]. Furthermore, this property of the migration is not different significantly between the lateral and distal or the forward and the rear regions, contrary to previous assumptions [30], [34]. The average speed was calculated to be 1.17±1.40 µm/min (n = 452 nuclei), and the average ratio of actual migration length to linear displacement over 30 minutes was 2.98±1.13 (n = 220 nuclei).

## Discussion

Visualizing and understanding cell behavior in the whole mouse embryo during gastrulation has been a long-standing goal in developmental biology. In this study, we developed the techniques of digital scanned light-sheet microscopy (DSLM) combined with mouse embryo culture system and computational analyzing tools for 3D tracking. Using these techniques, we performed live imaging of whole mouse embryos during gastrulation and analyzed the migrations of epiblast and mesodermal cells. Tracking of nuclear movements of the epiblast and mesoderm revealed that the bidirectional movement of INM is likely to be caused by active cell-autonomous forces rather than passive pushing forces from neighboring nuclei, and that mesodermal cells migrate individually rather than as a sheet. We also first report INM-like migration in the E5.5 epiblast, which is not yet pseudostratified.

We visualized a living mouse embryo during gastrulation till a depth of approximately 140 µm from the distal end. The resulting image showed stripe patterns and decreasing resolution along the optical axis (Figure 2A), which are shadows cast by refraction and scatter from the front region of the sample to the illumination light. These shadows can be reduced using multidirectional illumination techniques [39], [40]. Other light sheet techniques, such as structured illumination or multi-photon light sheet illumination, will also improve penetration when imaging mouse embryo during gastrulation.

The techniques we present here will be applicable to the study of other aspects of mouse development, for instance, migration of distal visceral endoderm, intercalation of definitive endoderm to visceral endoderm, and tracking of primordial germ cells. These topics are of significant interest, but have not yet been observed in live specimens because of the difficulty in live imaging of the mouse embryo. The phototoxicity problem still needs to be overcome to image the embryos for sufficient length of time: use of longer wavelength will be a solution in near future.

## Experimental Procedures

### Mice

Histone H2B-GFP and -mCherry transgenic mice were obtained from T. Fujimori (National Institute for Basic Biology) [41], [42]. Heterozygous embryos from intercrosses between wild-type ICR and Histone H2B-GFP (Figure 2, 3, 4, S2) or H2B-mCherry (Figure 5) were used. All animal experiments were carried out along the guidelines and the approval of The Institutional Animal Care and Use Committee of National Institutes of Natural Sciences (Permit Number: 10A087, 10A90).

### Embryo culture and imaging conditions

Mouse embryos were dissected into phenol red–free Dulbecco's modified Eagle's medium (DMEM, Invitrogen) supplemented with 10% fetal bovine serum (Invitrogen), preserving a portion of Reichert's membrane at the ectoplacental cone. Embryos were moved to the pocket space of the specially designed embryo holder which has been filled with medium culture from window of the cylinder (Figure 1C). Embryos were set into the hole of the holder and then the holder was pushed until the end of the cylinder. The embryos stayed at the position without window in order to prevent exposure of the embryo to air. The cylinder and holder was set at the stage and inserted into the culture medium in the chamber. The cylinder is elongated due to the length of cylinder holder. Embryos were cultured at 37°C with 5% CO_2_ and 5% O_2_, in medium containing 40% phenol red–free DMEM, 10% FCS (Invitrogen), 50% rat serum, 100 µM Trolox (Cayman), 100 U/ml penicillin, and 100 µg/ml streptomycin (Invitrogen). Gas was mixed by a GM-6000 from TOKAIHIT and blown into the air gap of the chamber. The sample holder was attached to the sample positioning system, which comprises three linear translation stages (M-111.2DG, Physik Instrumente) and one micro-rotation stage (M-116DG, Physik Instrumente). Three-dimensional image stacks were acquired every 1.5 or 3 min with a 300 ms exposure time per image and a z-spacing of 2.58 µm.

### Microscopy

We used a Digital Scanned Laser Light Sheet Fluorescence Microscope (DSLM) that was modified from our previously reported implementation [8]. An argon-krypton laser (35 LTL 835–200, Melles Griot) was used as the light source. The wavelength of the laser beam (488 or 568 nm) was selected using an acousto-optical tunable filter (AA.AOTF.nC-400–650 nm-PV-TN, AA Opto-Electronic) and scanned through the sample using a two-axis high-speed scan head (VM500+, GSI Lumonics). The scanned light sheet was created with an f-theta lens (S4LFT0061/065, Sill Optics) and a low-NA objective lens (Plan-Apochromat 5×/0.16, Carl Zeiss). Fluorescence emitted from probes was detected using a water immersion lens (Achroplan 20×/0.5, Carl Zeiss) and recorded with a CCD camera (Orca AG, Hamamatsu) through a long pass filter (RazorEdge RU 488 or 568, Semrock). The image data were recorded using our custom DSLM control software, which was developed in the Microsoft. NET framework. Illumination intensity was measured at the focal point of the illumination objective lens.

### Fixation and labeling

Dissected embryos were fixed and permeabilized overnight at 4°C in 4% paraformaldehyde in phosphate-buffered saline (PBS, Sigma) with 0.1% Triton X-100 (Sigma). After thorough washing, embryos were stained with Alexa Fluor 546 phalloidin (1∶100, Invitrogen) and 1 µM DRAQ5 (1∶1000, Biostatus) for one hour at room temperature.

### Pre-processing of image data and analysis of tracked epiblast and mesodermal cells

The acquired data were cropped and aligned in time and space with custom macros in ImageJ (National Institutes of Health). For tracking nuclei in epiblast, we used a custom-made assistant program for manual tracking, which records positions indicated by the user. For mesodermal cell tracking, we used another custom-made program that automatically track nuclei after the user points the position at first time point. The tracking results were confirmed by human eye, and wrong ones were deleted. Both tools were written in C++ (code available upon request). The automated tracking is based on an image recognition algorithm pointing the center of the nuclei. To reconstruct nuclear positions as 3D computer graphics shown in Figure 5 and Movie S4 to 8, we used POV-Ray (http://www.povray.org). Statistical analysis was performed in Microsoft Excel. Nuclear density in Figure 4B was calculated as the number of nuclei per 100 µm curve along the apical or basal surface of epiblast. The curve was drawn 8 µm from the apical or basal tips of the nuclei. The meddle was drawn at the center of the epiblast along its apical–basal axis. A nucleus was counted when the curve crossed the apical-basal axis at a point more than 20% of the axis length from the nearest end. The distance of the nearest-neighbor nuclei shown in Figure 4C was calculated as a distance between a nucleus and nearest nucleus in a cone having bottom of same spherical radius as the nucleus and height of twice radius at the nuclear center toward apical or basal side. Nuclear radius is set as 11 µm at apical side and 16.6 µm at basal side and it increases linearly along apical–basal axis. These values were measured with ImageJ (apical n = 30, basal n = 42). Classified regions of mesoderm in Figure 5 were defined according to the following criteria: where 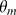 =  angle between right–left axis and proximal–distal axis, ‘lateral’ was defined as 0°≤
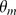
<60° or120°<
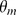
≤180°, and ‘distal’ was defined as 60°≤
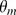
≤120°). Forward mesoderm was defined 30% region from front line at first time-point.

### Measuring method of three-dimensional cell division orientation

The 3D positions of the nuclei were determined using Imaris (Bitplane), and the velocities were calculated using Excel (Microsoft). The orientations of the nuclear divisions, 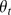, were calculated from the following equation using the projected orientations 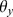 and 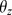 in the planes parallel and perpendicular to the proximal–distal axis (Figure S4). Each angle was calculated between the line along the epithelial surface and the line along the direction of division of the nuclei at anaphase.
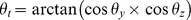



The directions were sub-divided into three groups with identical curved surface areas, parallel (0°≤
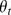
<19.5°), oblique (19.5°≤
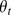
<41.8°), and perpendicular (41.8°≤
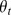
≤90°).

## Supporting Information

Figure S1 **Comparison of conventional and light sheet-based fluorescence microscopy (A).** (top) Optical paths of excitation (Ex) and detection (Det) in a conventional fluorescence microscope. Illumination and detection axes are parallel, and fluorophores outside the in-focus region are excited, resulting in low contrast. (bottom) The excitation and detection paths in light sheet-based fluorescence microscopy are perpendicular to one another. Fluorophores are excited in a region that overlaps with the focal region of the detection system. Since the emitted fluorescence photons are collected in parallel for all pixels in the camera, LSFMs acquire images at high speed and with low illumination intensity. Good depth penetration is achieved due to the low numerical aperture used in the illumination sub-system.
(TIF)

Figure S2 **Effect of phototoxicity by illumination on the development of mouse embryos during gastrulation.** Top row shows section images of the embryos at first time point (E6.5) and bottom row at 10 hours later (E7). In control, the embryo normally developed in the microscope chamber without illumination. When the embyo was illuminated throughout culture for 10 hours, it exhibited abnormal growth. When illuminated for only fist 3 hours, the growth was indistinguishable from the control. Estimated illumination power per hour is 4.6 mJ.
(TIF)

Figure S3 **Quantitation of division orientations in the epiblast.** (A) Distribution of division orientation. Blue, pink, and red indicate divisions parallel, oblique, and perpendicular to the apical surface, respectively. A, anterior; P, posterior; L, left; R, right; D, distal; Pr, proximal. (B) Percentage of parallel, oblique, and perpendicular divisions relative to epithelial surface. Measuring procedure is as described for Fig. S4.
(TIF)

Figure S4
**Method for measuring the three-dimensional division orientation of epiblast nuclei.** (A) The distribution of directions of nuclear divisions in the epiblast was calculated in planes both parallel and perpendicular to the proximal–distal axis. The angle was measured between the line along the epithelial surface and the division axis at anaphase. (B) The orientation 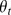 was calculated from the following equation using 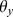 and 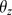. The measured division directions were pooled into three groups with identical curved surface areas: parallel (0°≤
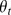
<19.5°), oblique (19.5°≤
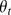
<41.8°), and perpendicular (41.8°≤
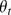
≤90°). 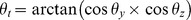.(TIF)

Movie S1 **Live imaging of Histone H2B–GFP mouse embryo at E6.5.** The optical section shown here is located about 78 µm from the distal end of the embryo. The time interval is 1.5 min. Gastrulation and mesodermal cell movement are clearly demonstrated; in the epiblast, interkinetic nuclear migration can be observed.
(MOV)

Movie S2 **Live imaging of mouse embryo at E6.** The optical section is located 65 µm from the distal tip of the embryo. The time interval is 3 min.
(MOV)

Movie S3 **Live imaging of mouse embryo at E5.5.** The optical section is located 40 µm from the distal tip of the embryo. The time interval is 3 min. Although the epiblast is not pseudostratified, INM-like movement occurs.
(MOV)

Movie S4 **Reconstructed epiblast nuclei from distal view.** Tracked positions were reconstructed as 3D computer graphics using POV-Ray (http://www.povray.org). Red, blue and green arrows indicate the anterior–posterior axis, right–left axis, and proximal–distal axis, respectively.
(MOV)

Movie S5 **Reconstructed epiblast nuclei from a lateral view.** Tracked positions were reconstructed as 3D computer graphic. Left side is shown. Red, blue, and green arrows indicate the anterior–posterior axis, right–left axis, and proximal–distal axis, respectively.
(MOV)

Movie S6 **Reconstructed trajectories of mesodermal cells.** Selected trajectories are shown on the embryo. The embryo consists of two surfaces: the outer visceral endoderm (blue) and a boundary surface between the visceral endoderm and mesoderm (green). Red semi-cylindrical shapes show the primitive streak, pointing in the posterior direction. Red, blue, and green arrows indicate the posterior–anterior axis, left–right axis, and proximal–distal axis, respectively.
(MOV)

Movie S7 **Time-lapse migrations of mesodermal cells.** Each mesodermal cell migrates, leaving a trail. The embryo consists of two surfaces: the outer visceral endoderm (blue) and a boundary surface between the visceral endoderm and mesoderm (green). Red semi-cylindrical shapes show the primitive streak, pointing in the posterior direction. Red, blue, and green arrows indicate the posterior–anterior axis, left–right axis, and proximal–distal axis, respectively.
(MOV)

Movie S8 **Time-lapse changes of triangles connected neighboring nuclei at the first time point.** Mesodermal cells are divided into two regions, lateral (cyan) and distal (magenta). The embryo consists of two surfaces: the outer visceral endoderm (blue) and a boundary surface between the visceral endoderm and mesoderm (green). Red semi-cylindrical shapes show the primitive streak, indicating posterior side. Red, blue, and green arrows indicate the posterior–anterior axis, left–right axis, and proximal–distal axis, respectively. As time goes by, the triangles become large and overlap each other, indicating that each cell migrates individually.
(MOV)

## References

[1] Tam PP, Gad JM, Kinder SJ, Tsang TE, Behringer RR (2001) Morphogenetic tissue movement and the establishment of body plan during development from blastocyst to gastrula in the mouse. BioEssays: news and reviews in molecular, cellular and developmental biology 23: 508–517. 10.1002/bies.1070 11385630

[2] Zernicka-Goetz M (2002) Patterning of the embryo: the first spatial decisions in the life of a mouse. Development 129: 815–829. 11861466 10.1242/dev.129.4.815

[3] Stern CD (2004) Gastrulation: from cells to embryo. Cold Spring Harbor, N.Y.: Cold Spring Harbor Laboratory Press. xvi, 731 p.

[4] Keller R (2005) Cell migration during gastrulation. Current opinion in cell biology 17: 533–541. 16099638 10.1016/j.ceb.2005.08.006

[5] Nowotschin S, Hadjantonakis AK (2010) Cellular dynamics in the early mouse embryo: from axis formation to gastrulation. Current opinion in genetics & development 20: 420–427. 20566281 10.1016/j.gde.2010.05.008 PMC2908213

[6] Yamanaka Y, Tamplin OJ, Beckers A, Gossler A, Rossant J (2007) Live imaging and genetic analysis of mouse notochord formation reveals regional morphogenetic mechanisms. Dev Cell 13: 884–896. 18061569 10.1016/j.devcel.2007.10.016

[7] Kwon GS, Viotti M, Hadjantonakis AK (2008) The endoderm of the mouse embryo arises by dynamic widespread intercalation of embryonic and extraembryonic lineages. Dev Cell 15: 509–520. 18854136 10.1016/j.devcel.2008.07.017 PMC2677989

[8] Keller PJ, Schmidt AD, Wittbrodt J, Stelzer EH (2008) Reconstruction of zebrafish early embryonic development by scanned light sheet microscopy. Science 322: 1065–1069. 18845710 10.1126/science.1162493

[9] Keller PJ, Stelzer EH (2008) Quantitative in vivo imaging of entire embryos with Digital Scanned Laser Light Sheet Fluorescence Microscopy. Curr Opin Neurobiol 18: 624–632. 19375303 10.1016/j.conb.2009.03.008

[10] Huisken J, Stainier DY (2009) Selective plane illumination microscopy techniques in developmental biology. Development 136: 1963–1975. 19465594 10.1242/dev.022426 PMC2685720

[11] Khairy K, Keller PJ (2010) Reconstructing embryonic development. Genesis. 10.1002/dvg.20698 21140407

[12] Guerrier S, Polleux F (2007) The ups and downs of neural progenitors: Cep120 and TACCs control interkinetic nuclear migration. Neuron 56: 1–3. 17920006 10.1016/j.neuron.2007.09.019

[13] Baye LM, Link BA (2008) Nuclear migration during retinal development. Brain Res 1192: 29–36. 17560964 10.1016/j.brainres.2007.05.021 PMC2674389

[14] Agathocleous M, Harris WA (2009) From progenitors to differentiated cells in the vertebrate retina. Annu Rev Cell Dev Biol 25: 45–69. 19575661 10.1146/annurev.cellbio.042308.113259

[15] Latasa MJ, Cisneros E, Frade JM (2009) Cell cycle control of Notch signaling and the functional regionalization of the neuroepithelium during vertebrate neurogenesis. Int J Dev Biol 53: 895–908. 19598111 10.1387/ijdb.082721ml

[16] Tao H, Suzuki M, Kiyonari H, Abe T, Sasaoka T, et al (2009) Mouse prickle1, the homolog of a PCP gene, is essential for epiblast apical-basal polarity. Proc Natl Acad Sci U S A 106: 14426–14431. 19706528 10.1073/pnas.0901332106 PMC2732806

[17] Tamai H, Shinohara H, Miyata T, Saito K, Nishizawa Y, et al (2007) Pax6 transcription factor is required for the interkinetic nuclear movement of neuroepithelial cells. Genes Cells 12: 983–996. 17825043 10.1111/j.1365-2443.2007.01113.x

[18] Del Bene F, Wehman AM, Link BA, Baier H (2008) Regulation of neurogenesis by interkinetic nuclear migration through an apical-basal notch gradient. Cell 134: 1055–1065. 18805097 10.1016/j.cell.2008.07.017 PMC2628487

[19] Zhang X, Lei K, Yuan X, Wu X, Zhuang Y, et al (2009) SUN1/2 and Syne/Nesprin-1/2 complexes connect centrosome to the nucleus during neurogenesis and neuronal migration in mice. Neuron 64: 173–187. 19874786 10.1016/j.neuron.2009.08.018 PMC2788510

[20] Norden C, Young S, Link BA, Harris WA (2009) Actomyosin is the main driver of interkinetic nuclear migration in the retina. Cell 138: 1195–1208. 19766571 10.1016/j.cell.2009.06.032 PMC2791877

[21] Sauer FC (1936) The interkinetic migration of embryonic epithelial nuclei. Journal of Morphology 60: 1–11.

[22] Fujita S (1960) Mitotic pattern and histogenesis of the central nervous system. Nature 185: 702–703. 13825588 10.1038/185702a0

[23] Miyata T (2008) Development of three-dimensional architecture of the neuroepithelium: role of pseudostratification and cellular ‘community’. Dev Growth Differ 50 Suppl 1S105–112. 18070110 10.1111/j.1440-169X.2007.00980.x

[24] Meyer EJ, Ikmi A, Gibson MC (2011) Interkinetic nuclear migration is a broadly conserved feature of cell division in pseudostratified epithelia. Current biology: CB 21: 485–491. 21376598 10.1016/j.cub.2011.02.002

[25] Minobe S, Sakakibara A, Ohdachi T, Kanda R, Kimura M, et al (2009) Rac is involved in the interkinetic nuclear migration of cortical progenitor cells. Neuroscience research 63: 294–301. 19367791 10.1016/j.neures.2009.01.006

[26] Schenk J, Wilsch-Brauninger M, Calegari F, Huttner WB (2009) Myosin II is required for interkinetic nuclear migration of neural progenitors. Proceedings of the National Academy of Sciences of the United States of America 106: 16487–16492. 19805325 10.1073/pnas.0908928106 PMC2752599

[27] Tsai JW, Lian WN, Kemal S, Kriegstein AR, Vallee RB (2010) Kinesin 3 and cytoplasmic dynein mediate interkinetic nuclear migration in neural stem cells. Nature neuroscience 13: 1463–1471. 21037580 10.1038/nn.2665 PMC3059207

[28] Kosodo Y, Suetsugu T, Suda M, Mimori-Kiyosue Y, Toida K, et al (2011) Regulation of interkinetic nuclear migration by cell cycle-coupled active and passive mechanisms in the developing brain. The EMBO journal 30: 1690–1704. 21441895 10.1038/emboj.2011.81 PMC3101991

[29] Davidson BP, Tam PP (2000) The node of the mouse embryo. Current biology: CB 10: R617–619. 10996084 10.1016/s0960-9822(00)00675-8

[30] Kinder SJ, Tsang TE, Wakamiya M, Sasaki H, Behringer RR, et al (2001) The organizer of the mouse gastrula is composed of a dynamic population of progenitor cells for the axial mesoderm. Development 128: 3623–3634. 11566865 10.1242/dev.128.18.3623

[31] Robb L, Tam PP (2004) Gastrula organiser and embryonic patterning in the mouse. Seminars in Cell & Developmental Biology 15: 543–554. 15271300 10.1016/j.semcdb.2004.04.005

[32] Lewis SL, Tam PP (2006) Definitive endoderm of the mouse embryo: formation, cell fates, and morphogenetic function. Developmental dynamics: an official publication of the American Association of Anatomists 235: 2315–2329. 16752393 10.1002/dvdy.20846

[33] Kwon GS, Viotti M, Hadjantonakis AK (2008) The endoderm of the mouse embryo arises by dynamic widespread intercalation of embryonic and extraembryonic lineages. Developmental cell 15: 509–520. 18854136 10.1016/j.devcel.2008.07.017 PMC2677989

[34] Nakatsuji N, Snow MH, Wylie CC (1986) Cinemicrographic study of the cell movement in the primitive-streak-stage mouse embryo. Journal of embryology and experimental morphology 96: 99–109. 3805991

[35] Kim SK, Shindo A, Park TJ, Oh EC, Ghosh S, et al (2010) Planar cell polarity acts through septins to control collective cell movement and ciliogenesis. Science 329: 1337–1340. 20671153 10.1126/science.1191184 PMC3509789

[36] Theveneau E, Marchant L, Kuriyama S, Gull M, Moepps B, et al (2010) Collective chemotaxis requires contact-dependent cell polarity. Developmental cell 19: 39–53. 20643349 10.1016/j.devcel.2010.06.012 PMC2913244

[37] Arboleda-Estudillo Y, Krieg M, Stuhmer J, Licata NA, Muller DJ, et al (2010) Movement directionality in collective migration of germ layer progenitors. Current biology: CB 20: 161–169. 20079641 10.1016/j.cub.2009.11.036

[38] Weber GF, Bjerke MA, DeSimone DW (2012) A mechanoresponsive cadherin-keratin complex directs polarized protrusive behavior and collective cell migration. Developmental cell 22: 104–115. 22169071 10.1016/j.devcel.2011.10.013 PMC3264825

[39] Huisken J, Stainier DY (2007) Even fluorescence excitation by multidirectional selective plane illumination microscopy (mSPIM). Opt Lett 32: 2608–2610. 17767321 10.1364/ol.32.002608

[40] Tomer R, Khairy K, Keller PJ (2011) Shedding light on the system: studying embryonic development with light sheet microscopy. Current opinion in genetics & development 21: 558–565. 21862314 10.1016/j.gde.2011.07.003

[41] Kurotaki Y, Hatta K, Nakao K, Nabeshima Y, Fujimori T (2007) Blastocyst axis is specified independently of early cell lineage but aligns with the ZP shape. Science 316: 719–723. 17446354 10.1126/science.1138591

[42] Abe T, Kiyonari H, Shioi G, Inoue K, Nakao K, et al (2011) Establishment of conditional reporter mouse lines at ROSA26 locus for live cell imaging. Genesis 49: 579–590. 21445964 10.1002/dvg.20753

