# Supplementary figures and images for: Live Imaging of Whole Mouse Embryos during Gastrulation: Migration Analyses of Epiblast and Mesodermal Cells
Source: PLoS One. 2013 Jul 8;8(7):e64506. doi: 10.1371/journal.pone.0064506 (PMC3704669; doi:10.1371/journal.pone.0064506)

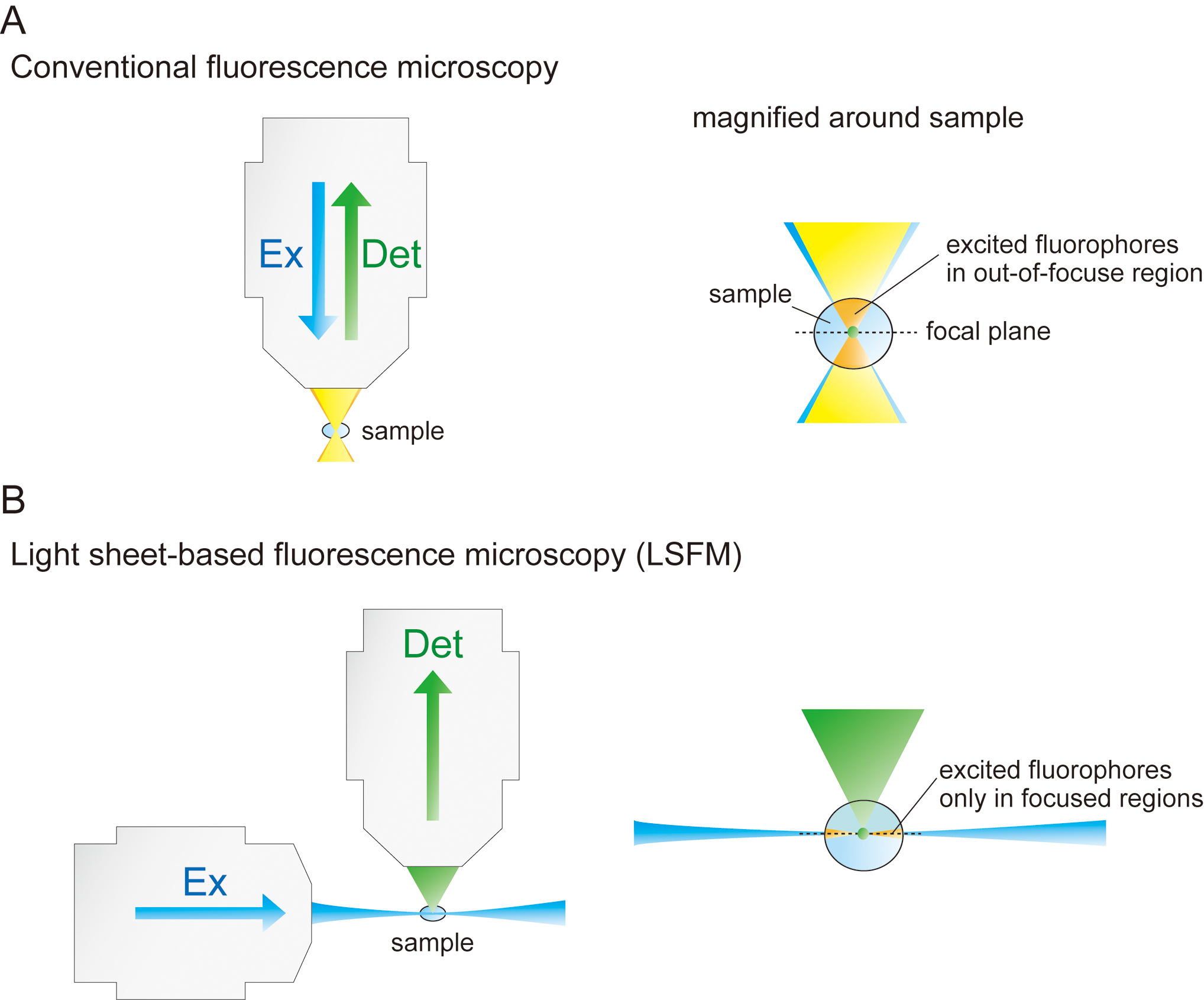

Supplement: Figure S1 — Comparison of conventional and light sheet-based fluorescence microscopy (A). (top) Optical paths of excitation (Ex) and detection (Det) in a conventional fluorescence microscope. Illumination and detection axes are parallel, and fluorophores outside the in-focus region are excited, resulting in low contrast. (bottom) The excitation and detection paths in light sheet-based fluorescence microscopy are perpendicular to one another. Fluorophores are excited in a region that overlaps with the focal region of the detection system. Since the emitted fluorescence photons are collected in parallel for all pixels in the camera, LSFMs acquire images at high speed and with low illumination intensity. Good depth penetration is achieved due to the low numerical aperture used in the illumination sub-system. (TIF) [file pone.0064506.s001.tif]

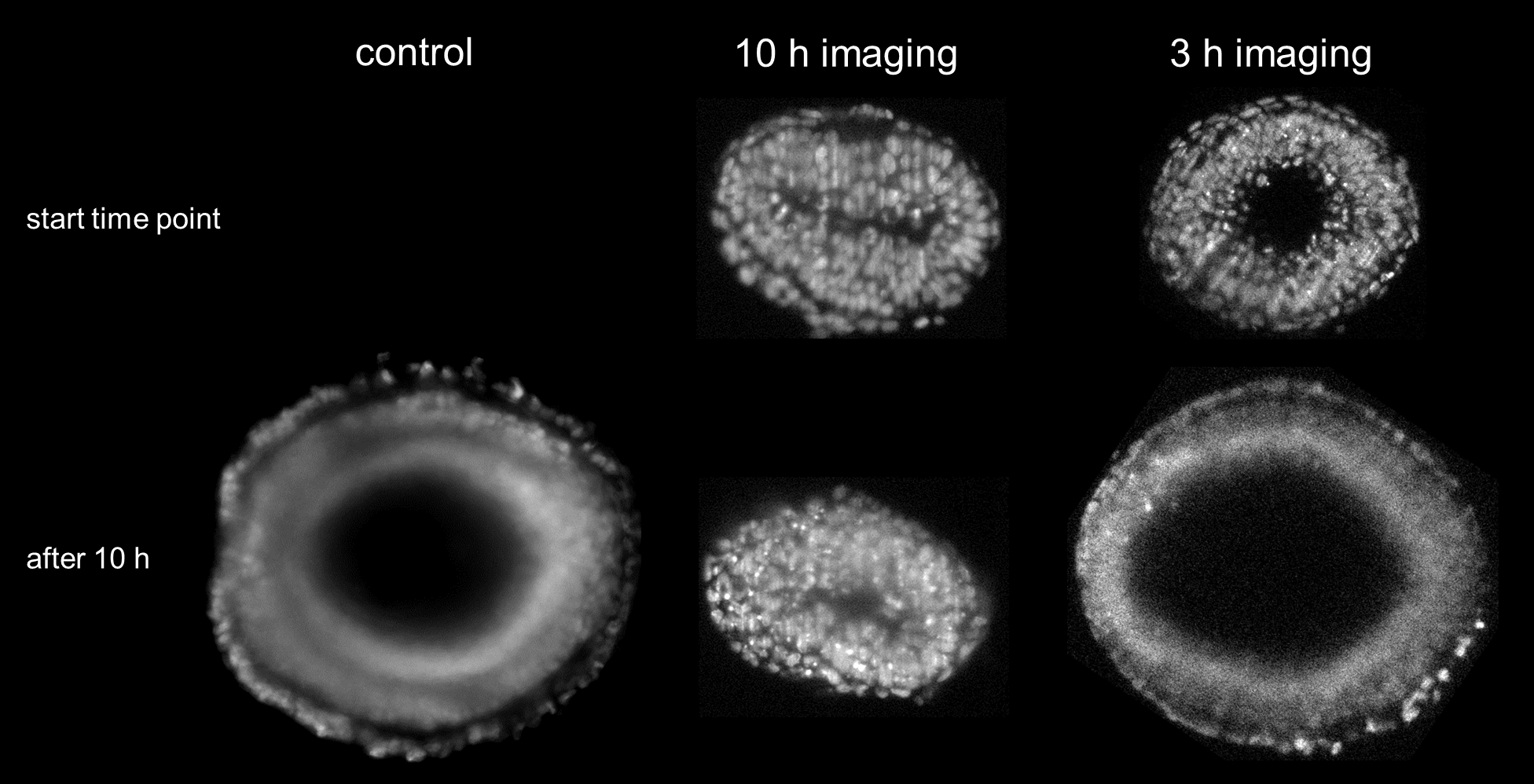

Supplement: Figure S2 — Effect of phototoxicity by illumination on the development of mouse embryos during gastrulation. Top row shows section images of the embryos at first time point (E6.5) and bottom row at 10 hours later (E7). In control, the embryo normally developed in the microscope chamber without illumination. When the embyo was illuminated throughout culture for 10 hours, it exhibited abnormal growth. When illuminated for only fist 3 hours, the growth was indistinguishable from the control. Estimated illumination power per hour is 4.6 mJ. (TIF) [file pone.0064506.s002.tif]

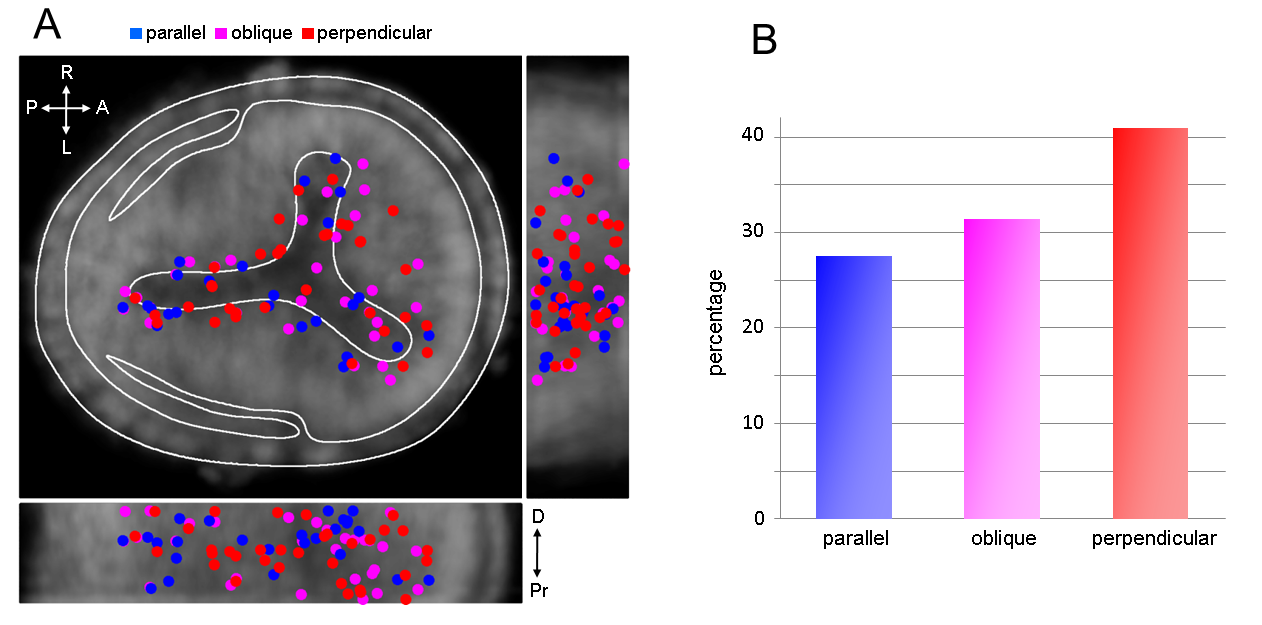

Supplement: Figure S3 — Quantitation of division orientations in the epiblast. (A) Distribution of division orientation. Blue, pink, and red indicate divisions parallel, oblique, and perpendicular to the apical surface, respectively. A, anterior; P, posterior; L, left; R, right; D, distal; Pr, proximal. (B) Percentage of parallel, oblique, and perpendicular divisions relative to epithelial surface. Measuring procedure is as described for Fig. S4. (TIF) [file pone.0064506.s003.tif]

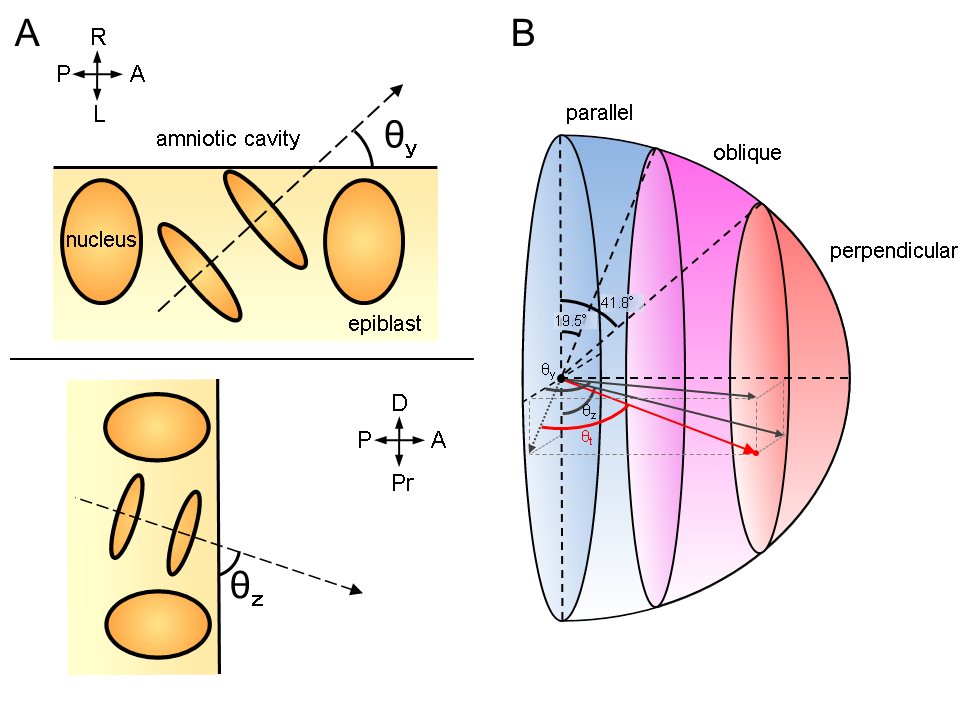

Supplement: Figure S4 — Method for measuring the three-dimensional division orientation of epiblast nuclei. (A) The distribution of directions of nuclear divisions in the epiblast was calculated in planes both parallel and perpendicular to the proximal–distal axis. The angle was measured between the line along the epithelial surface and the division axis at anaphase. (B) The orientation was calculated from the following equation using and . The measured division directions were pooled into three groups with identical curved surface areas: parallel (0°≤<19.5°), oblique (19.5°≤<41.8°), and perpendicular (41.8°≤≤90°). . (TIF) [file pone.0064506.s004.tif]
